# Comparative proteomics and glycoproteomics of plasma proteins in Indian visceral leishmaniasis

**DOI:** 10.1186/s12953-014-0048-z

**Published:** 2014-09-22

**Authors:** Arup Kumar Bag, Sutapa Saha, Shyam Sundar, Bibhuti Saha, Abhijit Chakrabarti, Chitra Mandal

**Affiliations:** Cancer Biology and Inflammatory Disorder Division, Council of Scientific and Industrial Research-Indian Institute of Chemical Biology, 4, Raja S.C. Mullick Road, Kolkata, 700 032 India; Crystallography & Molecular Biology, Saha Institute of Nuclear Physics, 1/AF Bidhannagar, Kolkata, 700 064 India; Department of Medicine, Institute of Medical Sciences, Banaras Hindu University, Varanasi, 221005 India; Department of Tropical Medicine, School of Tropical Medicine, Chittaranjan Avenue, Kolkata, 700073 India

**Keywords:** Visceral leishmaniasis, Plasma glycoproteomics, MARS column, M-LAC column, 2D-DIGE, MALDI-TOF/TOF mass spectrometry

## Abstract

**Background:**

Visceral leishmaniasis (VL) is a deadly parasitic diseases caused by *Leishmania donovani*; it is a major health problem in many countries. A lack of proper understanding of the disease biology, poor diagnostic methods and increasing drug resistance are the main reasons for the growing burden of VL infection. Comparative plasma proteomics are a relatively useful technique that can be used to investigate disease-associated alterations that can help in understanding host responses against pathogens, and might be useful in disease management and diagnosis.

**Result:**

In this study, a comparative proteomics and glycoproteomics approach using 2DE and 2D-DIGE was employed between early diagnosed VL patients of all age groups and healthy endemic and non-endemic controls in order to aid the recognition of disease-associated alterations in host plasma. Comparative proteomics was performed by the depletion of seven highly abundant plasma proteins. Comparative glycoproteomics was performed by the depletion of albumin and IgG, followed by purification of plasma glycoproteins using a multi lectin affinity column. From these two approaches, 39 differentially expressed protein spots were identified and sequenced using MALDI-TOF/TOF mass spectrometry. This revealed ten distinct proteins that appeared in multiple spots, suggesting micro-heterogeneity. Among these proteins, alpha-1-antitrypsin, alpha-1-B glycoprotein and amyloid-A1 precursor were up-regulated, whereas vitamin-D binding protein, apolipoprotein-A-I and transthyretin were down-regulated in VL. Alterations in the levels of these proteins in VL-infected plasma were further confirmed by western blot and ELISA.

**Conclusions:**

These proteins may be involved in the survival of parasites, resisting neutrophil elastase, and in their multiplication in macrophages, potentially maintaining endogenous anti-inflammatory and immunosuppressive conditions. Consequently, the results of this study may help in understanding the host response against *L.donovani*, which could help in the discovery of new drugs and disease management. Finally, these alterations on protein levels might be beneficial in improving early diagnosis considering those as biomarkers in Indian VL.

**Electronic supplementary material:**

The online version of this article (doi:10.1186/s12953-014-0048-z) contains supplementary material, which is available to authorized users.

## Introduction

Visceral leishmaniasis (VL), caused by *Leishmania donovani*, is a parasitic diseases that can be fatal if left untreated. Approximately 10 million people are affected currently and 350 million worldwide are known to be at risk, primarily in 88 endemic tropical, subtropical and Mediterranean countries. Amongst these countries, >90% of cases occur in India, Bangladesh, Nepal, Sudan and Brazil. Approximately 100,000 people become newly infected in India each year [1,2].

Owing to its complex manifestation and symptomatic resemblance, coupled with cross-reactivity with malaria and tuberculosis, diagnosis of VL is very problematic. Undiagnosed patients serve as a parasite reservoirs for disease transmission. Additionally, increasing rates of resistance against known drugs is becoming a problem. Consequently, considering the growing burden of VL, it is essential to better understand the disease biology and host responses against the pathogen, which could help in the discovery of new ways to manage the disease.

Plasma is the most logical sample to use in the identification of the disease. Owing to its physiological and pathophysiological importance, proteomic/glycoproteomic studies of plasma proteins provide significant insight into disease progression and pathology, as well as biomarker discovery and the identification of new drug targets for disease management [3,4]. Accordingly, alterations in VL plasma proteins were investigated by performing a comparative proteomic and glycoproteomic study using 2DE and 2D-DIGE between a large number of VL patients of all age groups, including children, with both endemic and non-endemic controls. The results revealed several VL-associated changes in the expression of glycoproteins and non glycosylated proteins in patient samples, which may have a role in disease progression and diagnosis.

## Results

### Reduction in the complexity of plasma proteins through the depletion of seven highly abundant proteins

**Figure 1:**
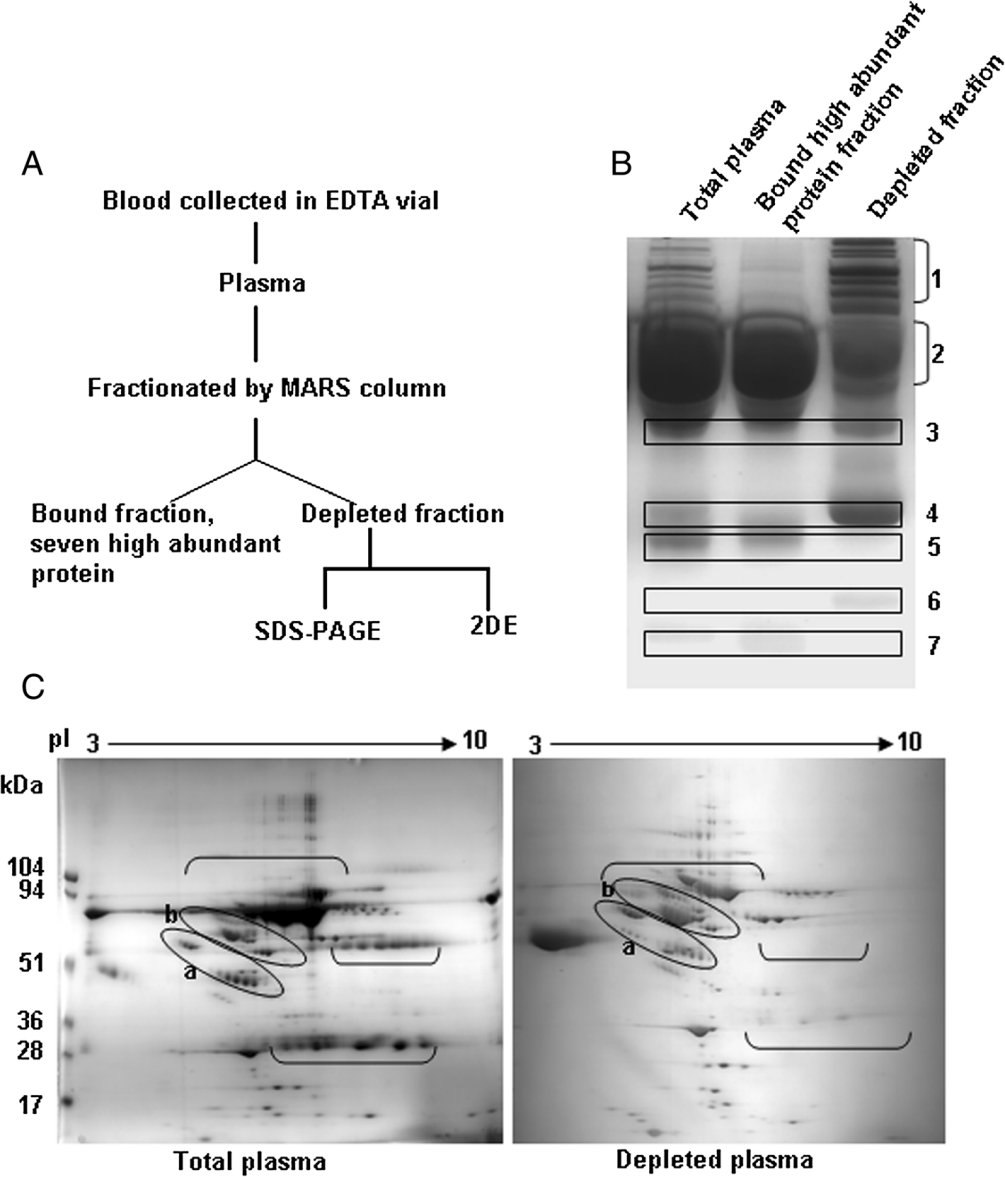
**MARS affinity column depleted high abundance proteins. A**. Flow diagram for depletion of seven high-abundance proteins from human plasma. **B**. Equal amounts (35 μg) of the total, bound and depleted plasma were loaded in the indicated lane, separated on a gradient SDS-PAGE gel (7.5-15%), and stained with coomassie blue. Positions 1, 3, 4 and 6 indicate enrichment after depletion, position 2 shows the appearance of comigrated bands in depleted fraction with high abundance proteins, whereas 5 and 7 positions represent decrease or removal after depletion. **C**. Total and depleted plasma (250 μg) were separated on 3–10 pI range IPG strip (17 cm) with 6-18% gradient gel and stained with coomassie blue. “ [ ” marked portion indicates depletion of proteins while alphabetically marked positions (a, b) indicate the appearance of some co-migrated spots after depletion.

When utilizing a comparative proteomics approach, sample-to-sample variations, diverse heterogeneity of glycoproteins and difference in protein abundance are some of the problems when studying disease-associated changes in plasma [3]. Therefore, to reduce the biological variations and dynamicity of different protein abundance, plasma samples were pooled and seven proteins of high abundance were subsequently depleted (Figure 1A). The results clearly illustrate the depletions at marked areas 2, 5 and 7, with enrichment visible at marked areas 1, 3, 4 and 6 in the depleted fraction (Figure 1B). A 2DE analysis of the crude and depleted plasma further demonstrated the depletions, enrichments and the appearance of new co-migrating low-abundance proteins (marked in Figure 1C). These results suggest that the dynamicity in the abundance of plasma proteins was lowered markedly, although a complete depletion was not achieved, with some high-abundance proteins remaining in compact associations with low-abundance proteins.

### Comparative proteomic analysis of depleted plasma samples demonstrated VL-associated alterations in 25 protein spots

**Figure 2:**
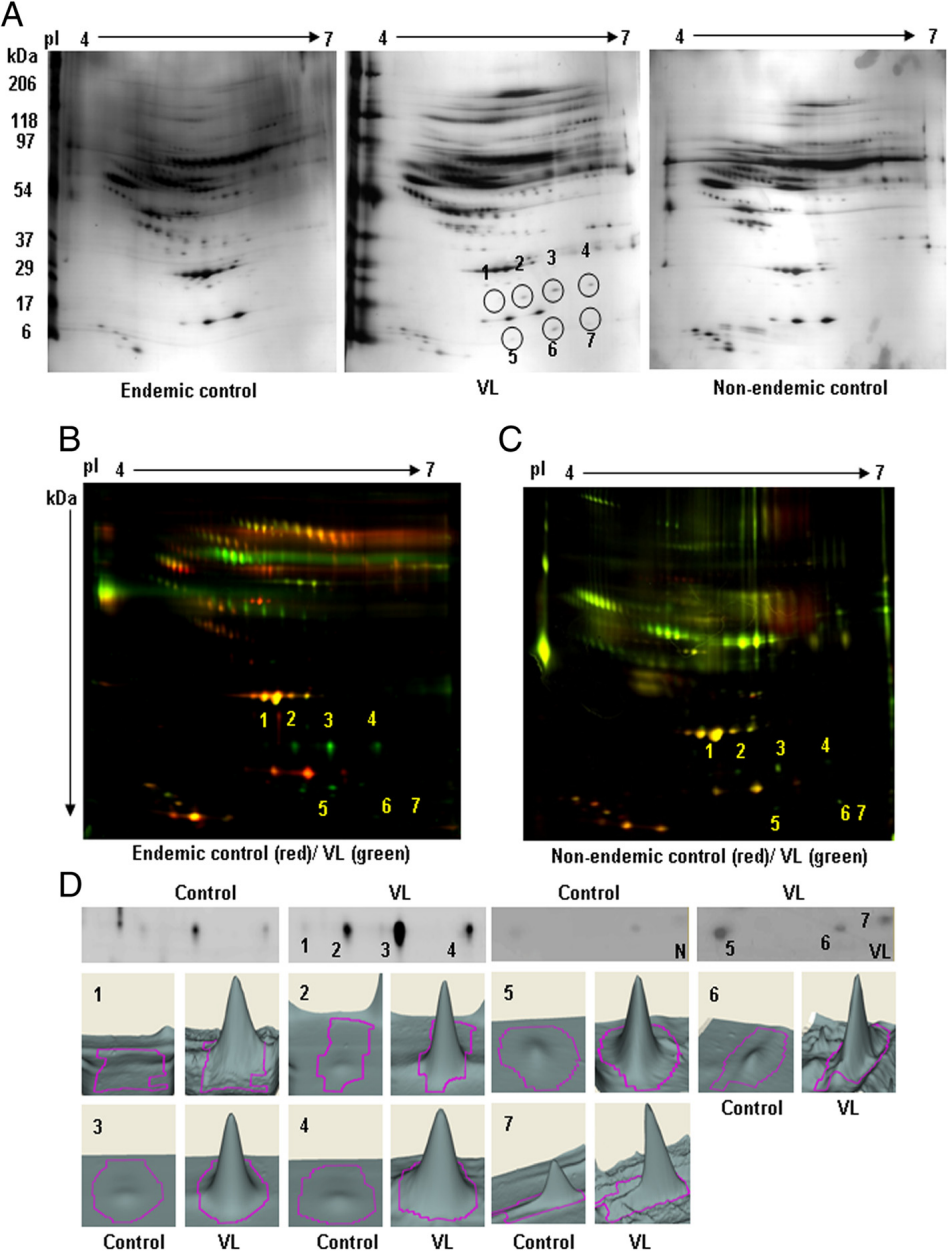
**Comparative 2DE and 2D-DIGE analysis in the acidic pI range. A**. Equal amount (50 μg) of depleted endemic control, VL and non-endemic control plasma samples were separated in 4–7 pI range IPG strip (17 cm) followed by SDS-PAGE (6-18%) and silver stained. Differentially expressed spots were circled with numbers after visual comparison between VL and endemic/non-endemic controls. **B**. **C**. Equal amount (50 μg) of depleted VL and endemic/non-endemic control plasma proteins labeled with CyDyes and separated. Images of VL and control were taken at different excitation/emission wavelengths and merged with ImageQuant Tool. Differentially expressed spots were marked by numbers following DIA analysis. **D**. Zoomed images and the analytic three dimensional expression profile of marked differently expressed spots 1–7.

The successful depletion of highly abundant plasma proteins from all pooled VL and control (endemic/non-endemic) samples prompted an investigation into whether these depleted plasma samples contain any changes in the expression of VL-associated proteins. Consequently, all depleted plasma samples were first resolved using 2D-PAGE within a 3–10 linear pI range, before being silver stained (Additional file 1). The results indicated that while the protein profile showed reproducibility, it was still difficult to compare protein spots owing to poor resolution across the large pI range. Subsequently, a 4–7 linear pI range was selected to provide better resolution and to specifically select only those spots that were up- or down-regulated in VL. By way of visual comparison, seven up-regulated spots indicated as 1–7 (Figure 2A) were identified in the VL samples in comparison with the endemic and non-endemic controls and therefore could be considered as VL-associated alterations. For further confirmation, plasma samples were analysed using a more sensitive 2D-DIGE with the same pI range. Differential-in-gel analysis (DIA) of VL against the endemic controls (Figure 2B) and VL against the non-endemic control (Figure 2C) revealed that the previously observed (Figure 2A) seven protein spots were significantly up-regulated in VL (marked as 1–7). Moreover, DIA was used to provide a three-dimensional analytic view (Figure 2D) of the magnitude of changes (Table 1, column 8–9, upper half) of the altered spots, and confirmed the VL-associated up-regulation of seven plasma proteins.

**Table 1:** **Identification of differentially expressed protein spots in VL**

**Spot no.**	**Protein name**	**Accession no**	**pI**	**MW in KDa**	**score**	**% of SC** ^***a***^	**VL/EC** ^***b***^	**VL/NEC** ^***b***^	**Expression**
**pI range 4-7**									
1	Unidentified	—	~6.5	~15	—	—	7.51	4.32	↑
2	Unidentified	—	~6.0	~15	—	—	27.08	18.31	↑
3	Unidentified	—	~5.5	~15	—	—	26.57	17.32	↑
4	Unidentified	—	~5.0	~15	—	—	18.82	15.39	↑
5	Amyloid A1 precursor	YLHUS	6.28	13.58	80	52	9.14	7.21	↑
6	Amyloid A1 precursor	YLHUS	6.28	13.58	89	53	8.04	6.10	↑
7	Unidentified	—	—	—	—	—	2.66	2.01	↑
**pI range 4.7-5.9**									
1	α-1-antitrypsin precursor	ITHU	5.37	46.88	230	36	2.63	2.12	↑
2	α-1-antitrypsin precursor	ITHU	5.37	46.88	240	49	7.49	6.32	↑
3	α-1-antitrypsin precursor	ITHU	5.37	46.88	230	36	11.29	9.21	↑
4	α-1-antitrypsin precursor	ITHU	5.37	46.88	250	54	7.41	6.37	↑
5	α-1-antitrypsin precursor	ITHU	5.37	46.88	230	36	5.58	5.60	↑
6	α-1-antitrypsin precursor	ITHU	5.37	46.88	392	55	4.30	4.50	↑
8	Vitamin-D binding protein	VYHUD	5.40	54.53	243	45	−3.94	−3.21	↓
9	Fibrinogen gamma-B chain precursor	FGHUGB	5.37	52.11	149	53	2.73	1.89	↑
10	Fibrinogen gamma-B chain precursor	FGHUGB	5.37	52.11	148	53	2.68	1.93	↑
11	APO A-I protein (frag.)	CAA00975	5.27	28.06	390	56	−3.31	−2.93	↓
12	Transthyretin	2ROYA	5.35	13.24	272	85	−3.96	−3.53	↓
13	Unidentified	—	~5.30	~30-45	—	—	4.93	3.97	↑
14	Unidentified	—	~5.30	~30-45	—	—	6.30	5.32	↑
15	Unidentified	—	~5.30	~30-45	—	—	5.60	4.73	↑
16	Unidentified	—	~5.30	~30-45	—	—	5.83	4.98	↑
17	Unidentified	—	~5.30	~30-45	—	—	4.88	4.54	↑
18	APO A-IV protein	LPHUA4	5.23	45.30	280	54	−4.82	−4.00	↓
19	APO A-I protein	CAA975	5.27	28.06	242	62	−2.68	−2.71	↓

Differentially expressed protein spots were identified by MALDI-TOF/TOF mass spectrometry. Combined MS and MS/MS results were analyzed by MASCOT and fold of up/down regulation determined from DIA analysis.

^*a*^SC- Sequence-coverage.

^*b*^Fold of increase and decrease ratio of the differentially expressed proteins from DIA analysis using DeCyder software from three different 2D-DIGE experiments with significant p < 0.05 were reported.

EC- endemic control.

NEC- non-endemic control.

↑- Up-regulation.

↓- Down-regulation.

**Figure 3:**
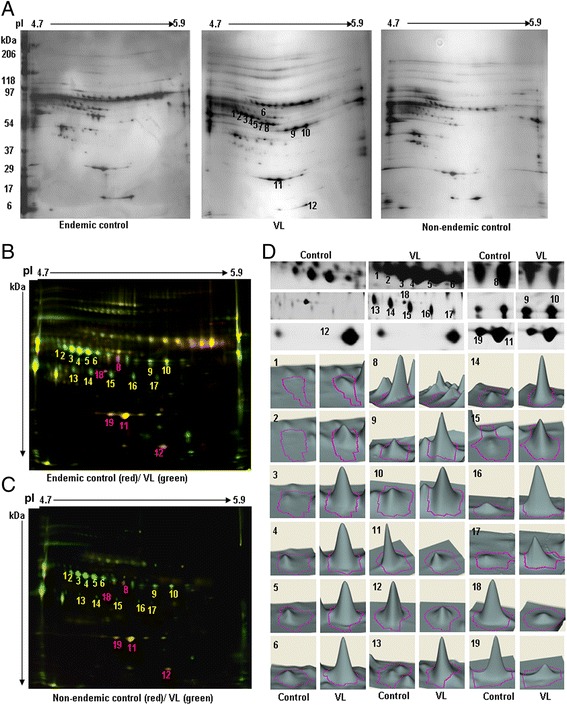
**VL-associated alterations in narrow acidic pI range. A**. Depleted VL and endemic/non-endemic control plasma proteins (50 μg) were focused using 4.7-5.9 pI range IPG strip (17 cm) and followed by SDS-PAGE (6-18%). Gels were silver stained and differentially expressed spots (1–12) were marked after visual comparison. **B**. **C**. Equal amounts (50 μg) of VL and endemic/non-endemic control were labeled by CyDyes with internal standard and separated as A. Fluorescent images were taken at different excitation/emission wavelengths and marked by numbers after DIA analysis. **D**. The magnified images and three dimensional expression profiles of the differentially expressed spots compared to controls were reported.

To resolve the higher molecular weight regions, the samples were again separated using 2D-PAGE through further narrowing the pI range (4.7–5.9) and a visual comparison revealed that the eight proteins present in spots 1–6, and 9 and 10 were up–regulated, whereas four proteins in spots 7, 8, 11 and 12 were down-regulated in VL (Figure 3A).

Additionally, DIA analysis after 2D-DIGE revealed 18 protein spots that were significantly differentially expressed in VL (Figure 3B,C). Among these proteins, the previously identified 1–12 protein spots were the same and expressed in a similar manner as was found in 2DE (Figure 3A). However, spot 7 remained unchanged following 2D-DIGE analysis. Interestingly, seven new protein spots were identified, among which 13–17 were up-regulated, and 18 and 19 were down-regulated significantly. Moreover, three dimensional expression profiling (Figure 3D) of the level of changes in expression (Table 1 column 8–9, lower half) from DIA confirmed their association with VL.

### Glycoproteomics revealed differential expression of the fourteen VL-associated glycoprotein spots

**Figure 4:**
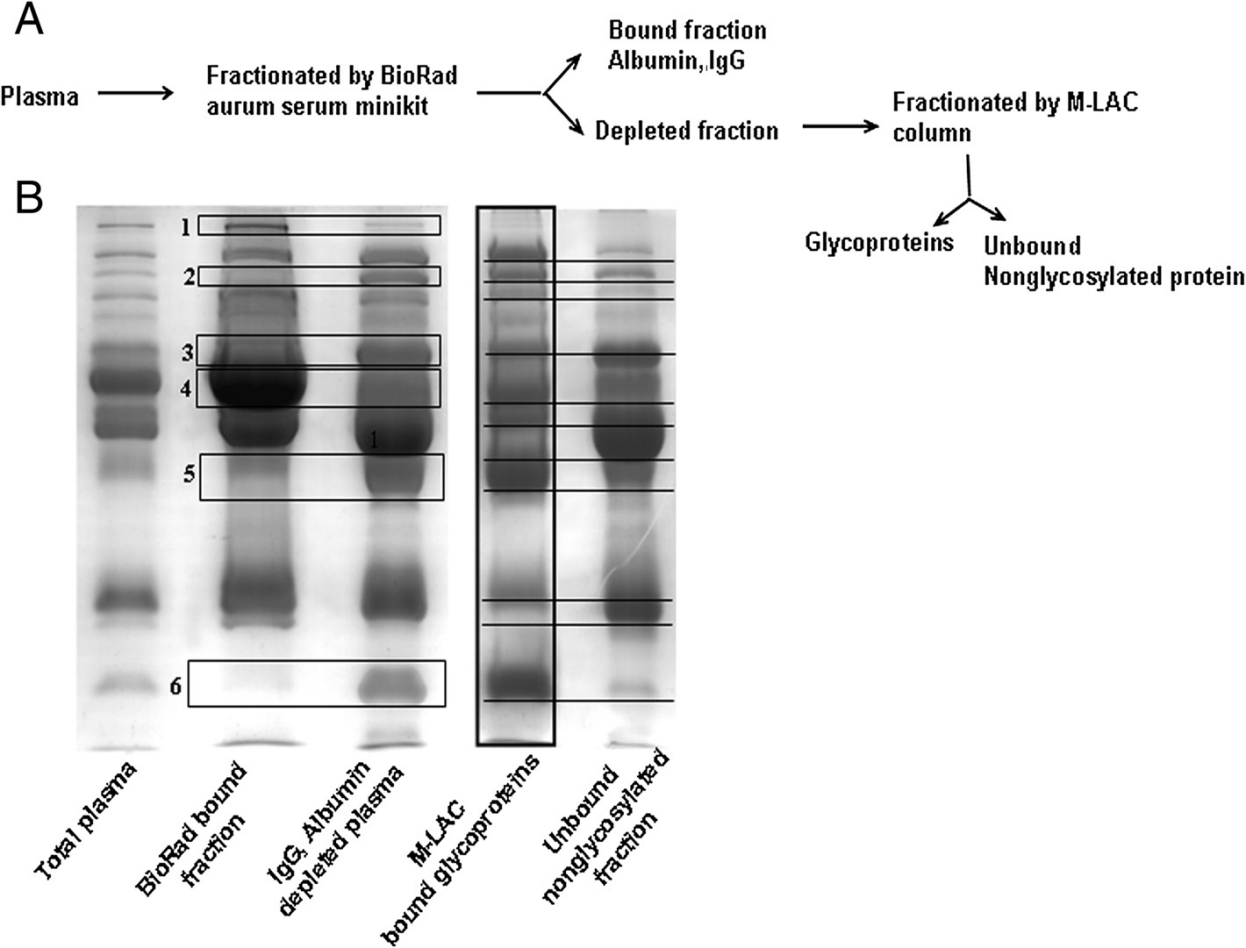
**Purification of glycoproteins by M-LAC column from albumin and IgG depleted plasma. A**. Brief flow diagram of glycoprotein purification from plasma samples. **B**. Equal amount (25 μg) of all fractions except total plasma (10 μg) was separated using 7.5-15% SDS-PAGE and stained with coomassie blue. Boxes 1 and 2 indicate the reduction whereas boxes 2, 3, 5 and 6 indicate the enhancement after depletion. Underlined bands clearly showed good binding of glycoproteins as well as the depletion of non-glycosylated proteins.

Glycosylation of proteins plays an important role in various biological processes including the immune response and cellular regulation [5]. Consequently, the possibility that any plasma glycoproteins were differentially expressed in VL was investigated. To achieve this, the most abundant non-glycosylated (albumin) and glycosylated (IgG) proteins were first depleted from plasma samples, as illustrated in Figure 4A, to avoid their interference in glycoprotein purification. Following this, glycoproteins were purified from the depleted plasma samples using multi-lectin affinity chromatography (M-LAC), illustrated in Figure 4A. Bound and unbound fractions from both columns were resolved using SDS-PAGE (Figure 4B). A comparison between lanes 2 and 3 demonstrated depletions at the first and fourth boxed positions and enrichments at the second, third, fifth and sixth portions in the depleted fraction. After this, using the lane 3 fraction, glycoproteins were purified as an M-LAC-bound fraction (lane 4) and non-glycosylated proteins were removed as an M-LAC-unbound fraction (lane 5).

**Figure 5:**
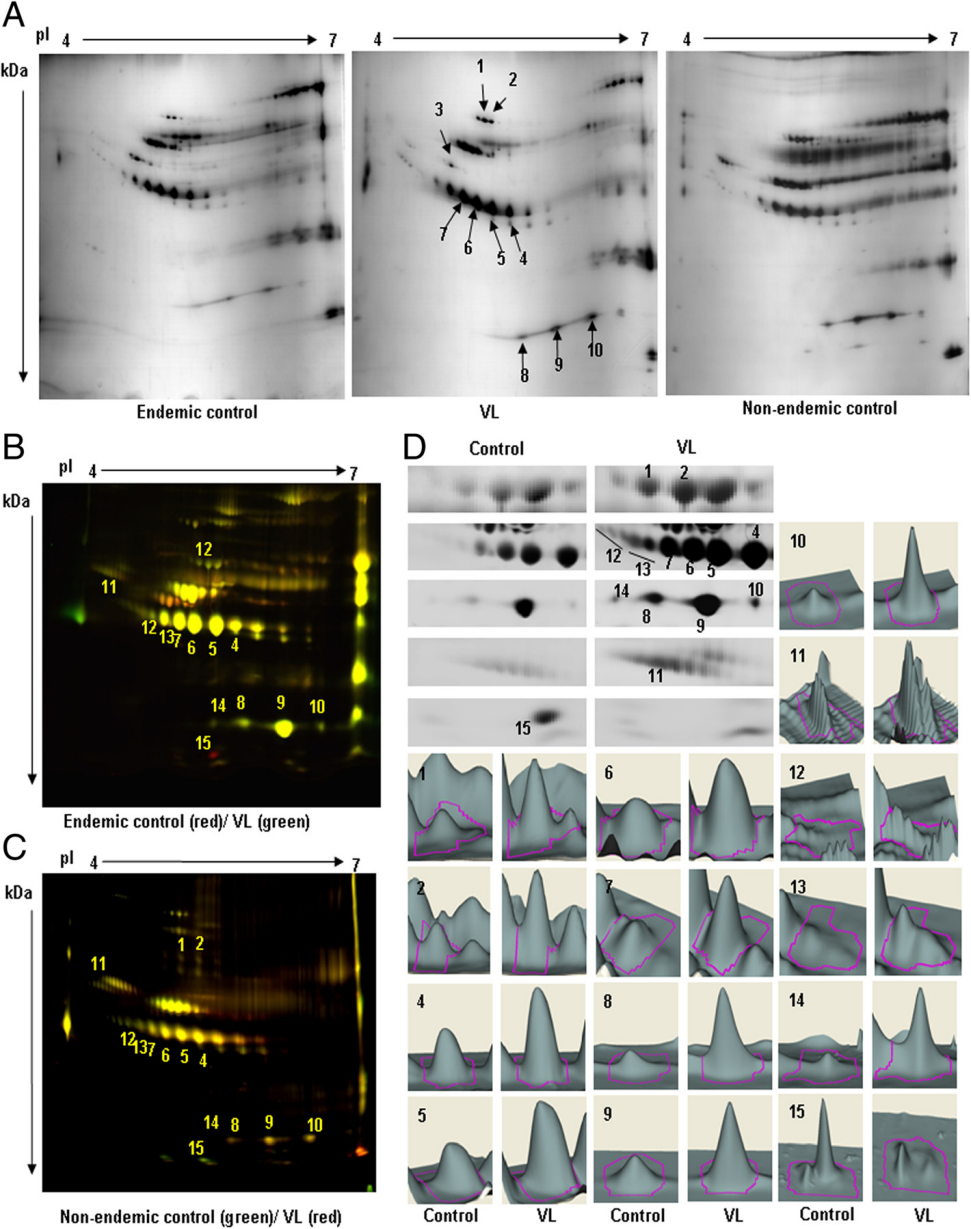
**Comparative glycoproteomics using 2DE and 2D-DIGE analysis. A**. Equal amounts (50 μg from each) from VL and controls (endemic/non-endemic) were separated using 4–7 linear pI range IPG strip (17 cm) and SDS-PAGE (6-18%). The gels were silver stained and differential expressions of VL glycoproteins compared to both endemic and non-endemic control were marked with an arrow and number after visual comparison. **B**. **C**. Purified glycoproteins (50 μg) from VL and endemic/non-endemic controls were labeled with CyDyes with corresponding internal standard and separated as A. Gels were scanned in different excitation/emission wavelengths and Cy3 and Cy5 images were merged using ImageQuant tool. Differently expressed spots were marked by numbers. **D**. Zoomed images of differentially expressed spots and 3D expression profile of the same after DIA analysis.

Sialic acids present in most of the glycoproteins make them acidic. Therefore, purified glycoproteins were separated using 2DE across a 4–7 pI range. Ten spots (marked numerically and with arrows) were differentially expressed in VL in comparison with endemic and non-endemic controls. Among these, nine spots (1, 2 and 4–10) were up-regulated and spot 3 was down-regulated (Figure 5A). For further confirmation, samples were again resolved using 2D-DIGE and were analysed using DIA analysis. The comparative analysis between VL and endemic control, and VL and non-endemic control indicated that 15 spots (marked numerically) were significantly differentially expressed in VL (Figure 5B,C). Among these, spots 1 and 2, and 4–10 were the same and were expressed similarly as was found using 2DE (Figure 5A), while spot 3 remained unchanged. Furthermore, the glycoproteins present in the newly detected spots 11–14 were up-regulated and 15 was down-regulated in VL; this result was corroborated using DIA expression profiles (Figure 5D, Table 2, columns 8 and 9). In summary, the glycoproteomic analysis revealed that fourteen protein spots were differentially expressed in VL.

**Table 2:** **Identification of VL associated differentially expressed glycoprotein spots**

**Spot no.**	**Protein name**	**Accession no**	**pI**	**MW in KDa**	**score**	**% of SC** ^***a***^	**VL/EC** ^***b***^	**VL/NEC** ^***b***^	**Expression**
1	α_1_-B glycoprotein	OMHU1B	5.65	52.48	112	34	2.24	2.01	↑
2	α_1_-B glycoprotein	OMHU1B	5.65	52.48	123	38	2.06	2.00	↑
4	Unidentified	—	~6.1	~38	—	—	2.01	1.92	↑
5	Haptoglobin precursor, allele-1	HPHU1	6.13	38.94	105	34	2.18	2.30	↑
6	Haptoglobin precursor, allele-1	HPHU1	6.13	38.94	157	34	2.39	2.49	↑
7	Haptoglobin precursor, allele-1	HPHU1	6.13	38.94	152	34	2.76	2.02	↑
8	Haptoglobin precursor, allele-2	HPHU2	6.13	45.86	68	24	3.68	3.69	↑
9	Haptoglobin precursor, allele-2	HPHU2	6.13	45.86	98	21	3.13	2.79	↑
10	Haptoglobin precursor, allele-2	HPHU2	6.13	45.86	117	28	3.81	2.67	↑
11	Unidentified	—	~5.5	~53	—	—	2.24	2.00	↑
12	Unidentified	—	~6.1	~38	—	—	2.87	2.65	↑
13	Haptoglobin precursor, allele-1	HPHU1	6.13	38.94	138	37	3.43	2.57	↑
14	Unidentified	—	~6.1	~45	—	—	3.60	2.97	↑
15	Unidentified	—	~6.2	~25	—	—	−3.12	−2.78	↓

Differentially expressed glycoprotein spots were identified by MALDI-TOF/TOF mass spectrometry. Combined MS and MS/MS results were analyzed by MASCOT and fold of up/down regulation were determined from DIA analysis.

^*a*^SC- Sequence-coverage.

^*b*^Fold of increase and decrease ratio of the differentially expressed proteins from DIA analysis using DeCyder software from three different 2D-DIGE experiments with significant p < 0.05 were reported.

EC- endemic control.

NEC- non-endemic control.

↑- Up-regulation.

↓- Down-regulation.

### Identification of 39 differentially expressed VL-associated protein spots using MALDI-TOF/TOF analysis

VL-associated non-glycosylated and glycosylated protein spots were sequenced. Tables 1 and 2 reveal that, among 39 different VL-associated protein spots, a few proteins appeared in multiple spots, suggesting a degree of heterogeneity. Finally, amyloid-A1 precursor (SAA1), alpha-1-antitrypsin (A1AT or SERPINA1), fibrinogen gamma–B chain precursor, alpha-1-B-glycoprotein (A1BG) and haptoglobin precursor allele 1, 2 were identified as being up-regulated, and vitamin-D binding protein (VDBP or GC), transthyretin (TTR), apolipoprotein A-I (APOA1) and A-IV were identified as being down-regulated proteins in VL infection (Tables 1, 2).

### Interactions of differentially expressed proteins support the association with VL

**Figure 6:**
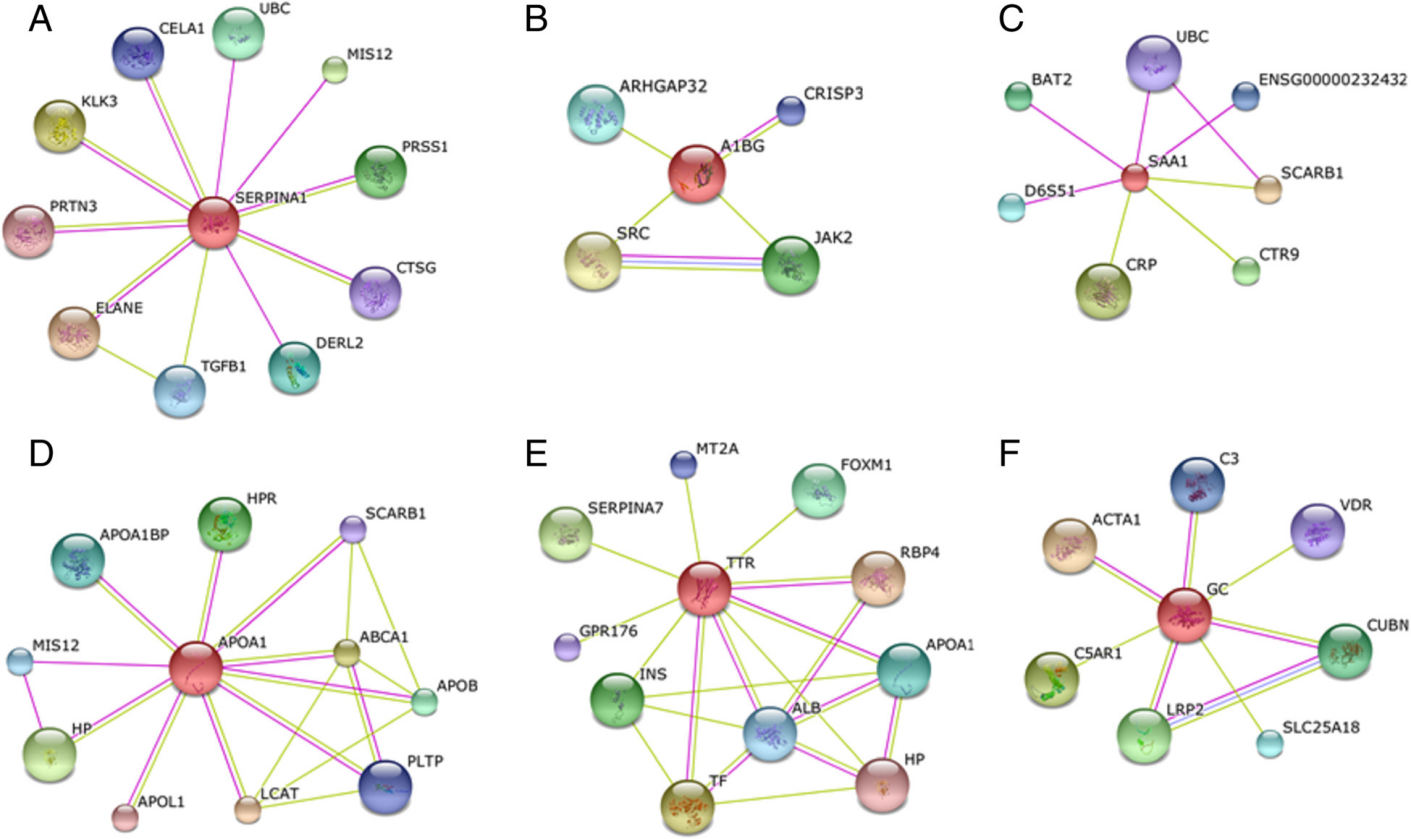
**Protein-protein interaction networks.** Protein-protein interactors of six differentially expressed protein **(A)** alpha-1-antitrypsin (SERPINA1), **(B)** alpha-1-B glycoprotein (A1BG), **(C)** amyloid A1 precursor (SAA1), **(D)** apolipoprotein A-I (APOA1), **(E)** transthyretin (TTR) and **(F)** vitamin-D binding protein (GC). The differentially expressed proteins indicated in the middle of every cluster with red and other interactors (gene names) with different colors. Purple and green colored lines for experimental and textmining evidence of different interactions.

The identification of protein–protein interaction networks in disease-associated proteins is an important way for develop system-level understanding of the cellular mechanisms of VL. Therefore, to speculate further on the probable biological-association and disease relevance, the interactions of these differentially expressed plasma proteins was examined. Protein–protein interaction analysis of the differentially expressed proteins revealed important connections of those proteins, through which they could exert their biological roles. Here the connections of six selected differentially expressed proteins that showed probable biological relevance in the context of *Leishmania* infection and disease progression from the existing evidence are highlighted (Figure 6 A–F, Additional file 2). The analysis demonstrated that SERPINA1 (A1AT) interacts with a wide variety of proteases and can inhibit their activity (Figure 6A). A1BG exhibited an interaction with cysteine-rich secretory protein 3 (CRISP3, Figure 6B). SAA1 interacts with scavenger receptor class B, member 1 (SCARB1), which maintains cholesterol balance between the cell surface and extracellular donors and acceptors (Figure 6C). APOA1 was found to interact with lecithin-cholesterol acyltransferase (LCAT), which is important for cholesterol reverse transport (Figure 6D). TTR demonstrated an interaction with iron transporter protein transferrin (TF, Figure 6E) and VDBP was found to interact with complement 5a receptor 1(C5AR1) and complement 3 (C3), which perform roles in the innate immune response (Figure 6F).

### Confirmation of the differential expression of six identified proteins by immunoblotting and ELISA

**Figure 7:**
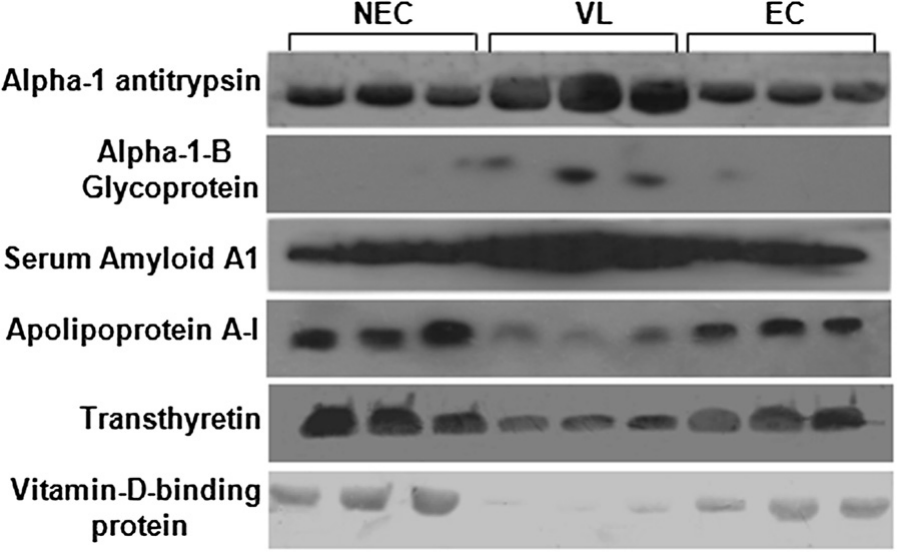
**Confirmation of differential expression of six proteins by western blot.** Western blot analysis illustrated the up-regulation of alpha-1-antitrypsin, alpha-1-B glycoprotein, Serum amyloid A1 and down-regulation of apolipoprotein A-I, transthyretin and vitamin-D binding protein in individual VL samples compared to endemic (EC) and non endemic controls (NEC).

Following this analysis, the differential expression status of six important proteins that might have a role in pathogenesis was checked using western blot analysis (Figure 7). The results revealed the up-regulation of alpha-1-antitrypsin, alpha-1-B glycoprotein and amyloid A1 precursor, and the down-regulation of vitamin-D binding protein, apolipoprotein A-I and transthyretin, which was previously observed using 2DE, 2D-DIGE and DIA analysis.

**Figure 8:**
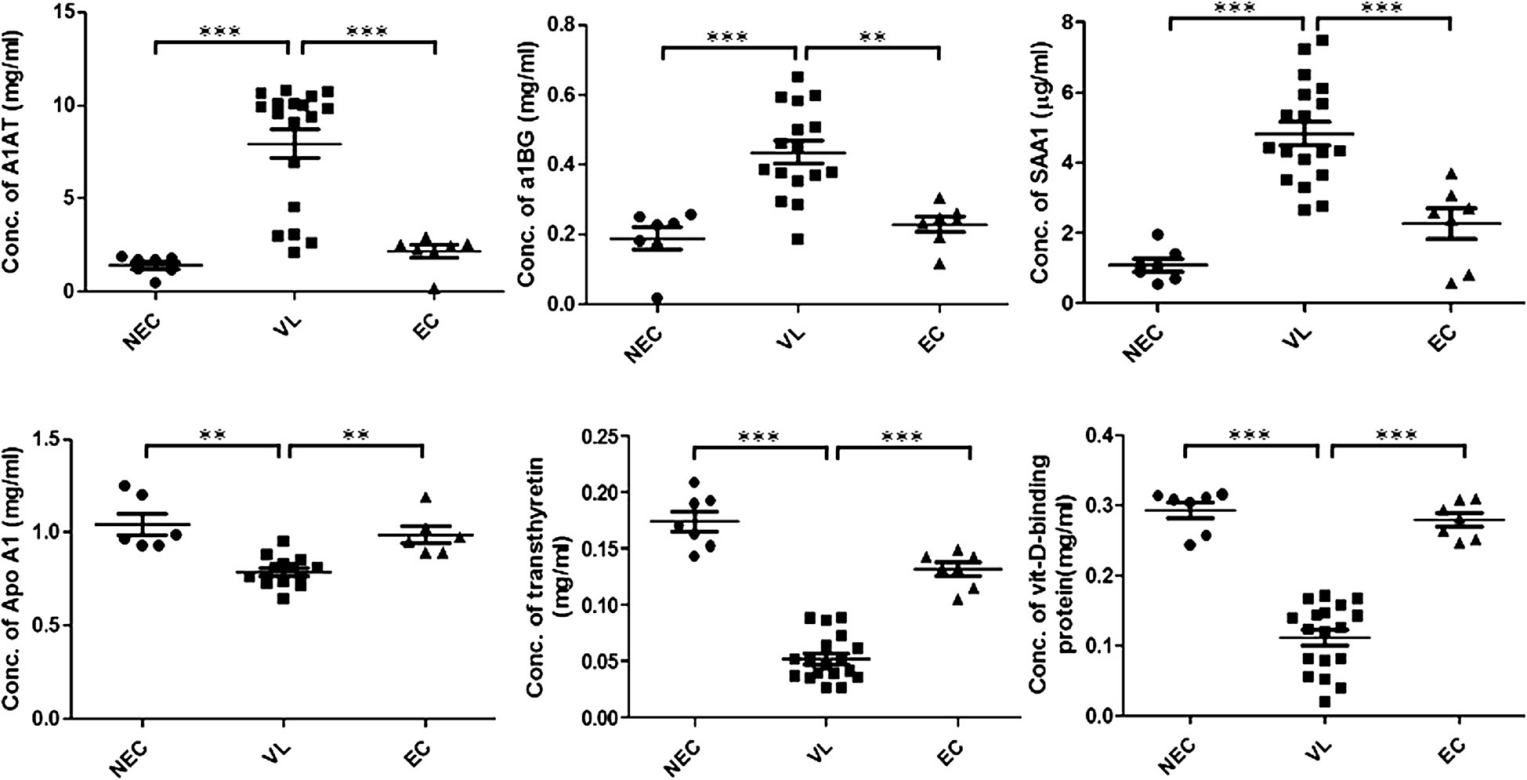
**Quantitative analysis of differentially expressed proteins.** Quantitative expression of differentially expressed six proteins, namely alpha-1-antitrypsin (A1AT), alpha-1-B glycoprotein (a1BG), amyloid A1 precursor (SAA1), apolipoprotein A-I (APOA1), transthyretin and vitamin-D binding protein in VL plasma in comparison to endemic (EC) and non endemic controls (NEC). A significance level of up or downregulation are indicated as “*” above the compared group.

Further, this was quantitatively verified by performing an ELISA using independent plasma, which also supported significant up- or down-regulation of these proteins (Figure 8, Additional file 3). In summary, it was concluded that these differentially expressed glycoproteins and non-glycoproteins were associated with VL across all age groups.

## Discussion

Since the dawn of proteomics, efforts have been made to identify and categorize the plasma proteins because of their potential utility as disease biomarkers, in therapeutic monitoring, and in understanding host responses to pathogens [3]. However, there are great challenges in using plasma for proteomic/glycoproteomic analysis because of their dynamic range of abundant proteins, diverse heterogeneity, the masking effects of highly abundant proteins, and the very low abundance of some important proteins. Therefore, the depletion of highly abundant proteins is essential in order to better visualise other important proteins. Accordingly, highly reproducible and competent MARS column and aurum serum mini kits were used. Glycoproteomics is a high-impact subfield of proteomics from the perspective of its biological and clinical significance. The type and structure of glycosylations of a secreted or membrane bound protein is highly specific depending on its physiological state and alter in different disease conditions [6]. Accordingly, the major findings of this study include the identification of VL-associated non-glycosylated and glycosylated proteins through comparative plasma proteomics/glycoproteomics, and the establishment of their probable biological relationship from protein–protein interaction network.

In this study, 2DE and quantitative 2D-DIGE proteomic technology was utilized. DIGE technology maximizes the probability of gel-to-gel reproducibility and the number of spots detected in comparison with conventional 2DE; it also provides reliable quantification. To minimize the complexity of biological variations of plasma, pooled samples of VL and endemic/non-endemic controls were used. Here, a total of ten differentially expressed (six up-regulated and four down-regulated) VL-associated proteins were identified across all age groups. Among these proteins, A1AT, A1BG and SAA1 were up-regulated, where as VDBP, TTR and APOA1were down-regulated. These VL-associated altered proteins may have the potential to serve as candidate biomarkers.

An interactome study revealed that A1AT interacts with a wide variety of proteases and can inhibit their activity; consequently, it helps to protect tissues from enzymes of inflammatory cells, in particular neutrophil elastase. As a result, over-expression of A1AT precursor may provide protection for a parasite from neutrophil elastase during the process of infection. Although the up-regulation of A1AT was reported only in infantile samples, this was confirmed in all VL patients irrespective of their ages [7].

A1BG, an *N*-glycosylated secreted plasma protein, is a member of the immunoglobulin superfamily; however, its function is unknown. Other than four glycosylation sites, *N*-acetylation and glycation are the two main post-translational modifications reported in this protein [8,9]. It is highly expressed in the adult and fetal liver however found in minute level in the blood, brain, lung, lymph node, ovary, testis and pancreas. Up-regulation of A1BG was observed in various types of cancer like pancreatic ductal carcinoma, liver and lung cancer [10-12]. In this study, A1BG was identified, for the first time, as being augmented in VL plasma. The study reveals that it interacts with CRISP3 and acts as a receptor to it [13]. As CRISP3 is expressed in neutrophils and is involved in the innate immune response, the A1BG–CRISP3 interaction may perform a role in immune suppression, which warrants further investigation.

The up-regulation of an acute phase protein, SAA1, is significant because it suggests an early stage of infection. SAA1 is a component of high-density lipoprotein (HDL) and can replace APOA1 from this complex. Consequently, this may be one of the reasons for APOA1 down-regulation. The result indicates that it interacts with SCARB1, a receptor of phospholipids, cholesterol esters and lipoproteins, and maintains the flux of free and esterified cholesterol between the cell surface and extracellular donors and acceptors. Macrophage membrane cholesterol plays an important role in parasite infectivity [14] so the up-regulation of SAA1–SCARB1 interactions may help in the development of VL infection.

Similarly, APOA1 was found to be down-regulated in VL patients in all age groups. It is the main component of HDL and is involved in the reverse transport of cholesterol from tissues to the liver for degradation, preventing its accumulation in macrophages through an interaction with LCAT. As such, the down-regulation of APOA1 may increases the level of macrophage cholesterol, which further assists parasite infectivity [14]. The down-regulation of these proteins was also observed in infants, indicating their VL-association [7,15].

TTR was also found to be down-regulated like earlier report [16]. TTR, a carrier of thyroxine and retinol, secreted by hepatocytes, may act as an endogenous anti-inflammatory mediator. It was observed that TTR interacts with iron-binding transport proteins (TF). Through modulating this interaction, a parasite might alter the host iron metabolism in favour of themselves, helping their proliferation within macrophages [17]. The result also indicated that an interaction occurs between TTR and APOA1, which is also down-regulated in VL plasma.

The interaction of VDBP with C5AR1 and C3 suggests that it has a role in inflammation, enhancing the chemotactic activity of complement 5a for neutrophils and activating macrophages [18], critical for the establishment of the early infection and subsequent propagation. Interactions with C3 may also modulate the innate immune response against parasites. Consequently, significant down-regulation of VDBP may delay the immune response against VL, thereby helping in the survival and multiplication of the parasite within macrophages. Taken together, all of these identified VL-associated proteins may provide a basic mechanism by which a parasite can survive in its host, although further investigation is required.

This study opens up new avenues for future exploration. Since VL is a poor man disease, therefore based on our result a simple and easily affordable diagnostic method using these differentially expressed proteins required to be developed to check in field conditions. Longitudinal study is also needed to evaluate their potential as prognostic marker. Additionally, distinct expression levels of A1AT in multiple spots demonstrates heterogeneity of glycoproteins, indicating an altered appearance of specific glycan structures in VL conditions. Glycan analysis to differentiate the structures present in those spots and discovering their roles will be the focus of future studies. One futuristic approach will be to develop a monoclonal antibody against that particular glycotope (spot 3 of A1AT), which may pave the way for better diagnostic tools for VL. While most of the highly abundant proteins were found to be depleted in this study, the identification of any changes in these proteins remains to be investigated. There are many connections in the protein–protein network other than those highlighted in relation to VL. Establishing the importance of these interactions requires further research.

## Conclusions

Comparative proteomic/glycoproteomic approaches within different sub-proteome levels and pI ranges helps to overcome the vast complexity of dynamic abundance and the heterogeneity of plasma proteins. This study illustrated significant alteration of A1AT, A1BG, SAA1, VDBP, APOA1 and TTR in VL plasma. In view of the growing burden of VL, these six proteins either independently or in combination may be useful as diagnostic/prognostic biomarkers and understanding parasite survival in the hostile environment of the host. Additionally, future experiments using particular glycotopes of A1AT may help in the early diagnosis of VL across all patient age groups, even when the parasite remains undetected in the spleen/bone-marrow.

## Methods

### Plasma samples

VL plasma samples of all age groups, including male and female, were collected from Kala-Azar Medical Research Centre (KMRC, n = 29) and School of Tropical Medicine (STM), India (n = 5) after confirmation of VL by microscopic demonstration of amastigotes in bone marrow/splenic aspirates, as per WHO recommendations [19]. Both endemic (n = 12) and non-endemic healthy control (n = 15) samples were included. To search disease-associated alterations that can help in understanding host responses against pathogens, and might be useful in disease management and diagnosis, those patients who had a low spleen parasitic load or for whom parasites were detected after a few days by in vitro splenic aspirate culture were selected (Table 3). Blood was collected using an EDTA vacutainer, incubated for 20–30 min at 4°C and centrifuged at 2000–2500 × g for 15 min. Plasma was separated and stored with a protease inhibitor at −80°C in small aliquots.

**Table 3:** **Clinical parameters of the sample population**

**Parameters**	**VL patients**	**Controls**	
		**Endemic**	**Non-endemic**
Individuals	34	12	15
Age range (Yr)	6-55	15-38	22-30
male: female	19:15	7:5	9:6
Child (≤10 yr): adult ratio	28:6	NA	NA
Duration of illness (days)^*a*^	60 ± 15	NA	NA
Splenic amastigote score^*a,b*^	1.6 ± 0.6	NA	NA
Spleen size (cm)^*a*^	5.37 ± 1.53	NA	NA
Hemoglobin conc (g/dl)^*a*^	8.1 ± 1.3	11.3 ± 1.2	12.4 ± 0.85
RBC count (10^6^/μl)^*a*^	2.95 ± 0.77	4.1 ± 1.1	5.1 ± 0.96

^*a*^Data are represented as mean ± SD.

NA- Not Applicable.

^*b*^Splenic amastigote score indicated as 4, >1 to 10 parasites/field; 3, >1 to 10 parasites/10 fields; 2, >1 to 10 parasites/100 fields; 1, >1 to 10 parasites/1,000 fields;

The study was approved by the Institutional Ethical Committee of Council of Scientific and Industrial Research- Indian Institute of Chemical Biology, Kolkata, India. The patient samples were collected from the Kala-Azar Medical Research Centre, Muzaffarpur, Institute of Medical Sciences, Banaras Hindu University, Varanasi and School of Tropical Medicine, Kolkata, India, with ethical approval from respective centres/institutes. The samples were collected with written informed consent from all participants/parents/guardians.

### Sample preparation

To reduce the complexity due to biological variation of samples and to have a reasonable sample size for two dimensional gel electrophoresis (2DE) and two dimensional differential in-gel electrophoresis (2D-DIGE) analysis, VL samples (n = 34) were reduced to seven pooled samples containing five different plasmas, except in the last (which contained four). Endemic (n = 12) and non-endemic (n = 15) controls were pooled separately to make four and five samples, respectively, containing three different plasma samples from each group (Table 3).

### Depletion of high abundance proteins

Seven high abundance proteins (albumin, transferrin, haptoglobin, antitrypsin, IgG, IgA and fibrinogen) were depleted from pooled plasma using a multiple affinity removal system HPLC column (4.6 × 100 mm, MARS) according to the manufacturer’s instructions (Agilent Technologies, USA) [20]. In brief, crude plasma (50 μL) was diluted five-fold with equilibration buffer A before being filtered through a 0.22-μm micro centrifuge filter and injected into the antibody column using an HPLC system (Shimadzu, Germany). The flow-through fractions from sequential injections were collected, pooled and concentrated to a volume of 200 μL using a 5-kDa MWCO spin concentrator; samples were stored in small aliquots at −80°C. The column was routinely regenerated by eluting bound high-abundance proteins with buffer B and, subsequently, the column was neutralized with buffer A before further use. The flow-through fraction was used for total proteome study.

### Isolation of plasma glycoproteins

Albumin and IgG were first depleted using the aurum serum protein mini kit (Bio-Rad, CA) [21]. Glycoproteins were purified from this depleted plasma using a multi lectin affinity chromatography (M-LAC) column by mixing 0.50 mL each of agarose-bound ConA, WGA and Jacalin lectin in a PD-10 disposable column (GE-Healthcare, USA). WGA binds to a terminal *N-*acetylglucosamine (dimer or trimer) attached to a common type of glycoprotein present in plasma. Similarly, Jacalin is another lectin and exhibits specificity towards *O*-glycosidically linked oligosaccharides containing galactosyl (β-1, 3) *N*-acetylgalactosamine (even in a mono or disialylated form). ConA recognizes the most commonly occurring sugar structure, α-linked mannose. Therefore, the combination of these three lectins would be expected to capture almost all types of commonly available *N* and *O*-linked glycoproteins present in the plasma. Depleted plasma (100 μL) was diluted to a volume of 1.0 mL with equilibrium buffer (20 mM Tris, 0.15 M NaCl, 1.0 mM MnCl_2_ and 1.0 mM CaCl_2_, pH 7.4) before being added to a pre-equilibrated column and incubated for 2 hr at a temperature of 4°C. The flow through fraction was collected as the non-glcosylated fraction. After washing three times with equilibration buffer, the bound fraction was eluted with 5 mL of elution buffer containing 20 mM Tris, 0.50 M NaCl, 0.20 M methyl-α-D-mannopyranoside, 0.20 M methyl-α-D-glucopyranoside, 0.50 M *N*-acetylglucosamine and 0.80 M galactose, pH 7.4. The bound fraction was concentrated using a 5-kDa MWCO spin concentrator before being stored as the purified glycoproteins in small aliquots at a temperature of −80°C for later use [22].

### Two dimensional gel electrophoresis (2DE), staining and imaging

Protein concentrations were estimated using a quick-start Bradford kit [23]. The required quantities of proteins were precipitated to remove interfering materials using a 2D-clean up kit (Bio-Rad) and resolubilised in rehydration buffer (7 M urea, 2 M thiourea, 2% CHAPS, 0.2% w/v ampholytes, 50 mM DTT and 0.004% bromophenol blue). An IPG-strip (Bio-Rad) of required pI/length was passively rehydrated with the resolubilised sample for 18 hr and focused using PROTEAN-IEF (Bio-Rad). The focused IPG-strips were equilibrated for 30 min each using equilibration buffer-I (6 M urea, 2 M thiourea, 0.375 M Tris–HCl, pH 8.8, 20% glycerol, 2% SDS, 0.005% bromophenol blue and 2% DTT) followed by buffer-II (2.5% iodoacetamide in place of DTT). The strips were then sealed on top of a gradient polyacrylamide gel with 0.5% agarose in electrophoresis buffer and separated using Bio-Rad gel apparatus [21,24].

Gels were silver stained for visualization and were subjected to MALDI-compatible colloidal coomassie/bio-safe coomassie staining for sequencing [25,26]. The gels were scanned using a Pro-Pic-II (Genomic solution, USA) image scanner and stored in 1% acetic acid at a temperature of 4°C.

### Two dimensional differential in-gel electrophoresis (2D-DIGE), imaging and analysis

Proteins were precipitated and resolubilised in DIGE-labelling buffer (7 M urea, 2 M thiourea, 4% CHAPS, 30 mm Tris–HCl, pH 8.5). Control, VL and internal standard samples (50 μg/15 μL) were labelled with three different CyDyes (GE-Healthcare) [27]. The pooled sample was rehydrated with rehydration buffer (7 M urea, 2 M thiourea, 2% CHAPS, 0.2% w/v ampholytes, 50 mm DTT) for 30 min using mild shaking in the dark. The mixed sample was resolved two dimensionally with minimal exposure to light.

The gel was scanned at three different excitation/emission wave lengths using Typhoon trio; images were then visualized and merged using ImageQuant tools (GE Healthcare). Differential expression patterns between the control and patient samples were statistically analysed using DeCyder v5.0 software (GE-Healthcare) by differential in-gel analysis (DIA). Spots from non-protein particles and background were filtered out and spots having a ≥2.0-fold increase or decrease with a p-value <0.05 were reported.

### Tryptic digestion and sequencing of protein spots by MALDI-TOF/TOF mass spectrometry

Protein spots were picked from the biosafe-coomassie-stained gel using a wide-bore tip head, and from fluorescent-stained gel using an automated spot picker (Pro-Pic-II). The excised gel pieces were destained and digested using the In-Gel-Digestion kit (Pierce) according to the manufacturer’s instructions. Concentrated peptides were desalted using ZipTip (Millipore) and eluted with 50% acetonitrile (4 μL) in 0.1% trifluoro-acetic acid (TFA).

Eluted peptides were spotted onto the MALDI target plate using the matrix-sample-matrix sandwich method using α-cyano-4-hydroxy cinnamic acid (5.0 mg/mL, Sigma) in 70% acetonitrile in 0.1% TFA. The mass spectrum of the digested peptides was measured using a 4700 MALDI-TOF/TOF analyser (Applied Biosystem, USA) in reflector mode. Before acquiring peptide mass spectrum from the sample, the system was calibrated using a standard 4700 Calmix (des-Arg 1-Bradykinine; 904.468, Angiotensin 1; 1296.685, Glu-1-fibrinopeptide B; 1570.677, ACTH 1–11; 2093.087, ACTH 18–39; 2465.199, ACTH 7–38; 3657.929). The mass spectrum was filtered between 800–4000 Da with a signal-to-noise ratio of 25 for generating the pick list using 4000 series explorer v3.5 software. To identify the acquired peptide spectrum, a search was performed using the parameters: Homo sapiens, maximum number of miss cleavage 1, mass tolerance 100 ppm, carbamidomethylation of cysteine, partial N-terminal acetylation, partial methionine oxidation and modification of glutamine as fixed modification of peptides. All the data were analysed using GPS explore v3.0 (TM) software and a search was performed of the combined MS and MS/MS results using the NCBInr, SWISS PORT and MSDB databases using MASCOT software v2.1 in order to identify the proteins. The identification was based on significant MASCOT Mowsescore (p < 0.05) and a comparison of observed versus expected pI and molecular weight from the 2DE gel.

### Protein-protein interaction analysis

An analysis of the protein–protein interactions of differentially expressed plasma proteins was performed using STRING v9.1 software. STRING is a database and web search tool of predicted and known protein–protein interactions. The physical (indirect) and functional (direct) interactions are considered when establishing the links, which are derived from genomic context, high-throughput experiments, co-expression analysis and previous literature resources. Here, experimental and textmining resources were used as a prediction method and the confidence score (≥0.700) as the parameter of interaction analysis. Nodes were coloured in the case of direct interaction with the input and white was used where it was absent. Two different size of the node reflect that there is structural information available for the protein (big, i.e., it is larger to fit the thumbnail picture) or not (small). Edges, the predicted functional links are the evidence of interactions between two proteins. Purple and green coloured edges represent the experimental and literature mining evidence of the interaction. The network is represented in confidence view, but action view was also used in the case of biological relevance analysis [28].

### Western blot analysis

Albumin and IgG depleted-plasma proteins (50 μg) were separated by SDS-PAGE (12%) and electro-transferred onto nitrocellulose membranes. The membrane was blocked using TBS-BSA and probed with a primary antibody overnight at a temperature of 4°C. After washing, the HRP-conjugated secondary antibody was added and the membrane was developed using the ECL system (Thermo scientific).

### Enzyme-linked immunosorbent assay (ELISA)

The expression status of the proteins of interest were quantitatively compared between the endemic control, non-endemic healthy control and VL plasma samples using an ELISA kit following the manufacturer’s supplied protocol. A separate ELISA kit was used for alpha-1-antitrypsin, amyloid A1 precursor, vitamin-D binding protein, apolipoprotein A-I_,_ transthyretin (Immunology Consultant Laboratory, Inc.) and alpha-1-B glycoprotein (Uscn Life Science Inc.). The sample population used for this quantitative analysis was a new set of independent samples and was different from those used for the proteomic analysis.

### Statistical analysis

Statistical analysis was performed using Graph Pad Prism 5 and Microsoft Excel software. The differences between the groups were analysed using at-test or Mann–Whitney *U*-test. Data represented in the tables were from at least three independent experiments with p < 0.05. In case of ELISA, standard error bars represent the standard error of the mean (±SEM) and significance of p value is represented as“***” (p < 0.001) or “**” (p < 0.01).

## Additional files

Additional file 1:
**Comparative 2DE between endemic control vs VL and non-endemic control vs VL.** Equivalent amount (300 μg) of MARS column high abundant protein depleted endemic control, VL and non-endemic control plasma were separated and processed as discussed in Figure 1C. Additional file 2:
**Predicted protein-protein interactors of six VL-associated proteins.**
Additional file 3:
**Sample population and quantitative ELISA data.** VL associated differential expression of plasma protein were quantitatively determined by ELISA in comparison to NEC and EC. Quantitative expression data of six proteins were represented as Mean ± SEM in three sets.

## Abbreviations

VL: Visceral Leishmaniasis
2DE: Two dimensional gel electrophoresis
2D-DIGE: Two dimensional differential in-gel electrophoresis
IEF: Isoelectric focusing
pI: Isoelectric point
IPG: Immobilized pH gradient
MALDI-TOF/TOF: Matrix assisted laser desorption ionization-time of flight/time of flight
M-LAC: Multi lectin affinity chromatography, MARS, multiple affinity removal system
MWCO: Molecular weight cut off
DTT: Dithiothreitol
TFA: Trifluoro-acetic acid
MS: Mass spectrometry
SDS: Sodium dodecyl sulphate
PAGE: Polyacrylamide gel electrophoresis
CHAPS: 3-[(3-cholamidopropyl) dimetylammonio]-1-propanesulfonate
