# Supplementary figures and images for: Comparative proteomics and glycoproteomics of plasma proteins in Indian visceral leishmaniasis
Source: Proteome Sci. 2014 Sep 22;12:48. doi: 10.1186/s12953-014-0048-z (PMC4179796; doi:10.1186/s12953-014-0048-z)

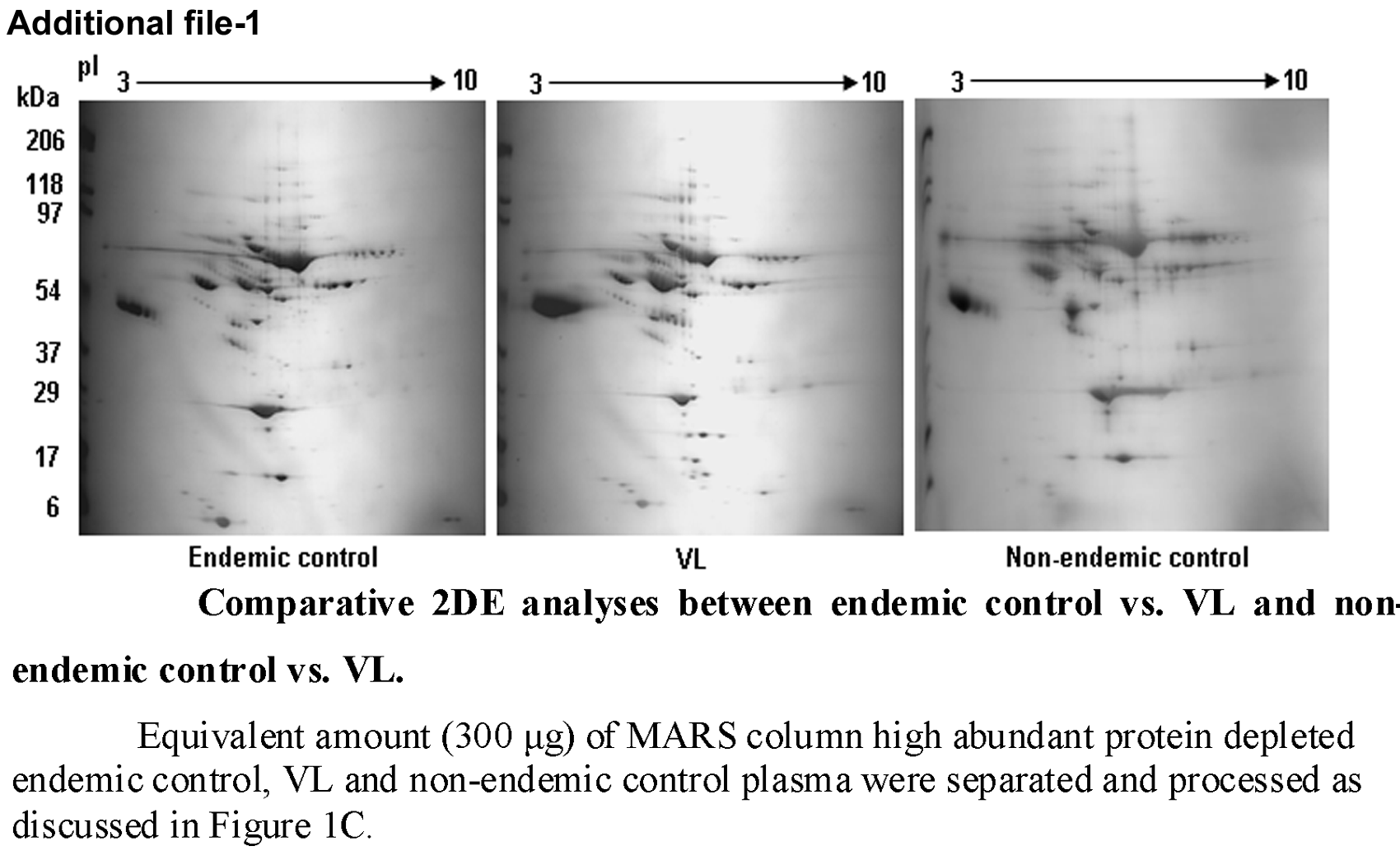

Supplement: Additional file 1: — Comparative 2DE between endemic control vs VL and non-endemic control vs VL. Equivalent amount (300 μg) of MARS column high abundant protein depleted endemic control, VL and non-endemic control plasma were separated and processed as discussed in Figure 1C. [file 12953_2014_48_MOESM1_ESM.tiff]
