# Supplementary material for: Comparative proteomics and glycoproteomics of plasma proteins in Indian visceral leishmaniasis
Source: Proteome Sci. 2014 Sep 22;12:48. doi: 10.1186/s12953-014-0048-z (PMC4179796; doi:10.1186/s12953-014-0048-z)
Supplement: Additional file 2: — Predicted protein-protein interactors of six VL-associated proteins. [file 12953_2014_48_MOESM2_ESM.doc]

**Additional file 2: Predicted protein-protein interactors of six VL-associated proteins**

| **Proteins** | **Predicted functional partners** | **Name and function** |
| --- | --- | --- |
| Alpha-1-antitrypsin (SERPINA1) | ELANE | Elastase, neutrophil expressed; Modifies the functions of natural killer cells, monocytes and granulocytes. Inhibits C5a-dependent neutrophil enzyme release and chemotaxis |
|  | KLK3 | Kallikrein-related peptidase 3; Hydrolyzes semenogelin-1 thus leading to the liquefaction of the seminal coagulum |
|  | MIS12 | MIS12, MIND kinetochore complex component, homolog (S. pombe); Part of the MIS12 complex which is required for normal chromosome alignment and segregation and for kinetochore formation during mitosis |
|  | PRSS1 | Protease, serine, 2 (trypsin 2); Has activity against the synthetic substrates Boc-Phe- Ser-Arg-Mec, Boc-Leu-Thr-Arg-Mec, Boc-Gln-Ala-Arg-Mec and Boc-Val- Pro-Arg-Mec. The single-chain form is more active than the two- chain form against all of these substrates |
|  | UBC | Ubiquitin C, depending of its attachment with lys residue plays different role. |
|  | DERL2 | Der1-like domain family, member 2; Functional component of endoplasmic reticulum-associated degradation (ERAD) for misfolded lumenal glycoproteins, but not that of misfolded nonglycoproteins. |
|  | TGFB1 | Transforming growth factor, beta 1; Multifunctional protein that controls proliferation, differentiation and other functions in many cell types. |
|  | CELA1 | Chymotrypsin-like elastase family, member 1; Acts upon elastin |
|  | CTSG | Cathepsin G; Serine protease with trypsin- and chymotrypsin-like specificity. Cleaves complement C3. |
|  | PRTN3 | Proteinase 3; Polymorphonuclear leukocyte serine protease that degrades elastin, fibronectin, laminin, vitronectin, and collagen types I, III, and IV (in vitro) and causes emphysema when administered by tracheal insufflation to hamsters |
| Alpha-1b glycoprotein (A1BG) | SRC | v-src sarcoma (Schmidt-Ruppin A-2) viral oncogene homolog (avian) |
|  | JAK2 | Janus kinase 2; Plays a role in leptin signaling and control of body weight (By similarity). Tyrosine kinase of the non-receptor type, involved in interleukin-3 and probably interleukin-23 signal transduction |
|  | ARHGAP32 | Rho/Cdc42/Rac GTPase-activating protein RICS. (RhoGAP involved in the beta-catenin-N-cadherin and NMDA receptor signaling)(Brain-specific Rho GTPase-activating protein)(GAB-associated Cdc42/Rac GTPase-activating protein) |
|  | CRISP3 | Cysteine-rich secretory protein 3, involved in innate immune response. |
| Serum amyloid A1 (SAA1) | SCARB1 | Scavenger receptor class B, member 1; Receptor for different ligands such as phospholipids, cholesterol ester, lipoproteins, phosphatidylserine and apoptotic cells. |
|  | CRP | C-reactive protein, pentraxin-related; Displays several functions associated with host defense |
|  | CTR9 | Ctr9, Paf1/RNA polymerase II complex component, homolog (S. cerevisiae) |
|  | BAT2 | HLA-B associated transcript 2; May play a role in the regulation of pre-mRNA splicing |
|  | D6S51 | HLA-B associated transcript-2 |
|  | ENSG  00000232432 | Novel protein |
|  | UBC | Ubiquitin C, depending of its attachment with lys residue plays different role. |
| Apolipoprotein  A1 (APOA1) | LCAT | Lecithin-cholesterol acyltransferase; Central enzyme in the extracellular metabolism of plasma lipoproteins. Among other substrates it esterifies the free cholesterol transported in plasma lipoproteins |
|  | ABCA1 | ATP-binding cassette, sub-family A (ABC1), member 1; cAMP-dependent and sulfonylurea-sensitive anion transporter. Key gatekeeper influencing intracellular cholesterol transport |
|  | HP | Haptoglobin; Haptoglobin combines with free plasma hemoglobin, preventing loss of iron through the kidneys and protecting the kidneys from damage by hemoglobin, while making the hemoglobin accessible to degradative enzymes |
|  | HPR | Haptoglobin-related protein |
|  | APOB | Apolipoprotein B (including Ag(x) antigen); Apolipoprotein B is a major protein constituent of chylomicrons |
|  | APOA1BP | Apolipoprotein A-I binding protein |
|  | MIS12 | MIS12, MIND kinetochore complex component, homolog (S. pombe); Part of the MIS12 complex which is required for normal chromosome alignment and segregation and for kinetochore formation during mitosis |
|  | PLTP | Phospholipid transfer protein; Converts HDL into larger and smaller particles. May play a key role in extracellular phospholipid transport and modulation of hdl particles |
|  | SCARB1 | Scavenger receptor class B, member 1; Receptor for different ligands such as phospholipids, cholesterol ester, lipoproteins, phosphatidylserine and apoptotic cells. |
|  | APOL1 | Apolipoprotein L, 1; May play a role in lipid exchange and transport throughout the body. May participate in reverse cholesterol transport from peripheral cells to the liver |
|  | RBP4 | Retinol binding protein 4, plasma; Delivers retinol from the liver stores to the peripheral tissues. In plasma, the RBP-retinol complex interacts with transthyretin, this prevents its loss by filtration through the kidney glomeruli |
|  | TF | Transferrin; Transferrins are iron binding transport proteins which can bind two Fe(3+) ions in association with the binding of an anion, usually bicarbonate. |
|  | SERPINA7 | Serpin peptidase inhibitor, clade A (alpha-1 antiproteinase, antitrypsin), member 7; Major thyroid hormone transport protein in serum |
|  | INS | Insulin; Insulin decreases blood glucose concentration. It increases cell permeability to monosaccharides, amino acids and fatty acids. It accelerates glycolysis, the pentose phosphate cycle, and glycogen synthesis in liver |
|  | FOXM1 | Forkhead box M1; Transcriptional activatory factor. May play a role in the control of cell proliferation |
|  | APOA1 | Apolipoprotein A-I; Participates in the reverse transport of cholesterol from tissues to the liver for excretion by promoting cholesterol efflux from tissues and by acting as a cofactor for the lecithin cholesterol acyltransferase (LCAT). |
|  | ALB | Albumin; Serum albumin, the main protein of plasma, has a good binding capacity for water, Ca(2+), Na(+), K(+), fatty acids, hormones, bilirubin and drugs. |
|  | MT2A | Metallothionein 2A; Metallothioneins have a high content of cysteine residues that bind various heavy metals; these proteins are transcriptionally regulated by both heavy metals and glucocorticoids |
|  | GPR176 | G protein-coupled receptor 176; Orphan receptor |
|  | HP | Haptoglobin; Haptoglobin combines with free plasma hemoglobin, preventing loss of iron through the kidneys and protecting the kidneys from damage by hemoglobin, while making the hemoglobin accessible to degradative enzymes |
| Vit-D binding  Protein (GC) | ACTA1 | Actin, alpha 1, skeletal muscle; Actins are highly conserved proteins that are involved in various types of cell motility and are ubiquitously expressed in all eukaryotic cells (By similarity) |
|  | C5AR1 | Complement component 5a receptor 1; Receptor for the chemotactic and inflammatory peptide anaphylatoxin C5a. This receptor stimulates chemotaxis, granule enzyme release and superoxide anion production |
|  | LRP2 | Low density lipoprotein-related protein 2; Acts together with cubilin to mediate HDL endocytosis (By similarity). May participate in regulation of parathyroid- hormone and para-thyroid-hormone-related protein release |
|  | CUBN | Cubilin (intrinsic factor-cobalamin receptor); Cotransporter which plays a role in lipoprotein, vitamin and iron metabolism, by facilitating their uptake. |
|  | SLC25A18 | Solute carrier family 25 (mitochondrial carrier), member 18; Involved in the transport of glutamate across the inner mitochondrial membrane. Glutamate is cotransported with H(+) |
|  | C3 | Complement component 3; C3 plays a central role in the activation of the complement system. |
|  | VDR | Vitamin D (1,25- dihydroxyvitamin D3) receptor; Nuclear hormone receptor. Transcription factor that mediates the action of vitamin D3 by controlling the expression of hormone sensitive genes. |
