# Supplementary material for: Comparative proteomics and glycoproteomics of plasma proteins in Indian visceral leishmaniasis
Source: Proteome Sci. 2014 Sep 22;12:48. doi: 10.1186/s12953-014-0048-z (PMC4179796; doi:10.1186/s12953-014-0048-z)
Supplement: Additional file 3: — Sample population and quantitative ELISA data. VL associated differential expression of plasma protein were quantitatively determined by ELISA in comparison to NEC and EC. Quantitative expression data of six proteins were represented as Mean ± SEM in three sets. [file 12953_2014_48_MOESM3_ESM.doc]

**Additional file 3:** **Sample population and quantitative ELISA data**

VL associated differential expression of plasma protein were quantitatively determined by ELISA in comparison to NEC and EC. Quantitative expression data of six proteins were represented as Mean ± SEM in three sets.

| **Protein** | **No. of Sample** | **Mean ± SEM** | | |
| --- | --- | --- | --- | --- |
|  |  | **NEC** | **VL** | **EC** |
| A1AT | NEC (n=7), EC (n=7), VL (n=18) | 1.395 ± 0.192 mg/ml | 7.926 ± 0.77 mg/ml | 2.151 ± 0.339 mg/ml |
| a1BG | NEC (n=7), EC (n=7), VL (n=16) | 0.189 ± 0.033 mg/ml | 0.435 ± 0.033 mg/ml | 0.228 ± 0.022 mg/ml |
| SAA1 | NEC (n=7), EC (n=7), VL (n=18) | 1.092 ± 0.18 μg/ml | 4.836 ± 0.342 μg/ml | 2.269 ± 0.435 μg/ml |
| ApoA1 | NEC (n=6), EC (n=6), VL (n=14) | 1.042 ± 0.059 mg/ml | 0.787 ± 0.021 mg/ml | 0.987 ± 0.045 mg/ml |
| Transthyretin | NEC (n=7), EC (n=7), VL (n=18) | 0.174 ± 0.009 mg/ml | 0.052 ± 0.005 mg/ml | 0.132 ± 0.006 mg/ml |
| Vit-D binding protein | NEC (n=7), EC (n=7), VL (n=18) | 0.292 ± 0.011 mg/ml | 0.111 ± 0.011 mg/ml | 0.279 ± 0.01 mg/ml |

EC - endemic control

NEC- non-endemic control

SEM- standard error of mean
